# Exome-wide comparative analyses revealed differentiating genomic regions for performance traits in Indian native buffaloes

**DOI:** 10.1080/10495398.2023.2277376

**Published:** 2023-11-07

**Authors:** Vishakha Uttam, Vikas Vohra, Supriya Chhotaray, Ameya Santhosh, Vikas Diwakar, Vaibhav Patel, Rajesh Kumar Gahlyan

**Affiliations:** Animal Genetics & Breeding Division, ICAR-National Dairy Research Institute, Karnal, Haryana, India

**Keywords:** Indian native buffalo breeds, performance traits, population genetics, whole-exome sequencing

## Abstract

In India, 20 breeds of buffalo have been identified and registered, yet limited studies have been conducted to explore the performance potential of these breeds, especially in the Indian native breeds. This study is a maiden attempt to delineate the important variants and unique genes through exome sequencing for milk yield, milk composition, fertility, and adaptation traits in Indian local breeds of buffalo. In the present study, whole exome sequencing was performed on Chhattisgarhi (n = 3), Chilika (n = 4), Gojri (n = 3), and Murrah (n = 4) buffalo breeds and after stringent quality control, 4333, 6829, 4130, and 4854 InDels were revealed, respectively. Exome-wide F_ST_ along 100-kb sliding windows detected 27, 98, 38, and 35 outlier windows in Chhattisgarhi, Chilika, Gojri, and Murrah, respectively. The comparative exome analysis of InDels and subsequent gene ontology revealed unique breed specific genes for milk yield (*CAMSAP3*), milk composition (*CLCN1, NUDT3*), fertility (*PTGER3*) and adaptation (*KCNA3, TH*) traits. Study provides insight into mechanism of how these breeds have evolved under natural selection, the impact of these events on their respective genomes, and their importance in maintaining purity of these breeds for the traits under study. Additionally, this result will underwrite to the genetic acquaintance of these breeds for breeding application, and in understanding of evolution of these Indian local breeds.

## Introduction

In India, there are 20 distinct registered buffalo breeds (*Bubalus bubalis*) flourishing under many agro-ecological conditions with various breeding, and management techniques.^1^ However, they probably vary in their agro-economic traits and adaptation to local environments. These breeds are settled in not so salubrious conditions under tropical and subtropical parts of the country experiencing hot and humid climatic conditions. These buffaloes catered to variety of specialized uses, including dairy, draft, meat, and manure.^2^ Each unique breed is likely to harbor genetic variants specific to above functions and to adaptation. These could be a valuable genetic resource for locating genes that are sustained under long-term natural selection.^3–5^ Such a diverse germplasm that has undergone an extensive adaptation to a wide range of agro-climatic conditions. This is believed to be result of accumulation of advantageous mutations through natural selection and survival of fittest. Exome-wide comparative analysis can be utilized to comprehend how these spontaneous mutations have modified the genetic structure of these populations over a long term.

Indian local and unique germplasm like Chhattisgarhi, Gojri, and Chilika breeds from India have not yet been explored fully. To date, only a few studies are reported in Murrah breed such as identification of genes for lactation and fertility traits using GWAS (*ANKRD44, CCSER1, GRIA3,* etc),^6^ several dairy candidate genes identification using ddRAD sequencing (*PLCE1* and *CACNB2*),^7^ and selection signature for milk production and immunity using ddRAD sequencing (*SLC37A1, PDE9A*, etc).^8^ Few studies in Chilika, Gojri and Chhattisgarhi buffalo have been done earlier by our group namely, comparative expression profiling studies of heat stress genes (*GLUT-1* and *HSP60*) in Chilika buffalo,^9^ polymorphic study on Exon-40 of *FASN* gene in Gojri and Chhattisgarhi.^10^

However, these native breeds have been reported to be strikingly different. This could be due to the difference in their utility and might have been selected accordingly. The Chhattisgarhi breed exhibits dual-purpose characteristics, evincing a daily yield of 4 kg of milk in hot and humid conditions, withstanding a low-input system. Nevertheless, in females there is delayed age at first calving that varies from 60 to 72 months.^11^ The "Bathaan system" is practiced during the paddy sowing period, in their breeding tract. Gojri is a multi-utility and semi-migratory breed that is primarily raised by the Gujjar community of Punjab and Himachal Pradesh. They are uniquely adapted to grazing and agricultural operations on hilly terrain due to their sure footing.^12^ The Chilika buffalo, an indigenous and insular breed, exhibits significant adaptability to brackish water and is geographically secluded.^13^ Reared under extensive management systems, these dual-purpose animals serve as a vital source of subsistence for farmers in the region, owing to their capacity to endure high temperatures and humidity. Average milk yield of Chilika buffalo is between 2-6 liters per day. The dairy products derived from their milk boast a commendable shelf life.^14^ Recent advances in next generation sequencing (NGS) technology and sequence analysis tools have allowed sequencing to become a feasible tool to explore potential variants underlying the above phenotypic traits in livestock populations.

Fast and inexpensive sequencing of exome and genome analysis makes population based genetic study an attractive option. These new techniques enable the investigator to evaluate genome-wide variation underlying performance attributes in buffalo populations. Further these tools aid in comparing the genetic makeup of these populations and cataloguing the change in frequencies of the selected alleles adapted to diverse climatic zones.^15^ Estimating the degree of genetic differentiation among populations would help to understand the genome and to make inferences about their evolutionary history which is also one of the objectives of population genetic studies.^16^^,^^17^ Population differentiation probably occurs due to long-term natural selection, migration, mutation, and genetic drift in a population offering them unique genetic structure. The most widely used population genetic statistical tools includes admixture analysis, principal components analysis (PCA),^18^ fixation index (F_ST_), nucleotide diversity (π) and Tajima’s D (TD) by utilizing the commonly used bioinformatical software.

One of the many sequencing tools, whole exome sequencing technique aims solitary on highly enriched exome part of the genome. Exome is best-characterized region that correlates to phenotypes as it explores highly interpretable allelic variants underlying the economic traits. According to various studies it can be concluded that exome data set is most suitable to execute evolutionary studies, as both recent and ancient genetic differences, change in allele frequencies, and identification of the functional variant or gene responsible for adaptation could be marked in a population.^19–25^ Functional variants like InDels can be precisely extracted from exome data set and are considered second best after single nucleotide polymorphisms-based data.^26^

As far as we are aware, no comprehensive whole exome analysis has been done so far to scrutinize the genetic structure and population differentiation in these local buffalo germplasms of India. It is hypothesized that the meticulous exploration of exome will uncover the large number of variants and candidate gene that have been naturally selected for their performance. This present study was conducted to explore population structure and differentiation under selection and to further explore insights into the biological mechanisms underlying these attributes specific to Chhattisgarhi, Chilika, Gojri, and Murrah breeds of buffalo using whole exome based sequencing technique.

## Material and methods

### Sample collection and whole-exome sequencing

In the present study, a total of four rare buffalo germplasm were taken, namely Chhattisgarhi (n = 3), Chilika (n = 4), Gojri (n = 3), and Murrah (n = 4) from their respective native breeding tracts. According to their geographical distribution, breeds under study are located into four different regions namely, central hilly and plateau region of Chhattisgarh (Chhattisgarhi), eastern-costal region of Odisha (Chilika), northern sub-Himalayan region (Gojri), and northern plain region of parts of Haryana (Murrah). For each breed, sampling was done ensuring that entire breeding tract area was covered. Pre-capture libraries were created from whole genomic DNA from 14 blood samples of buffalo by utilizing All Exon Bovine ((Agilent SureSelect (catalog no. 5067- 5582)) capture kit according to the manufacturer’s instructions. In-solution capture method was used for genomic DNA fragmentation, template preparation and exome capture. 150 × 2 bp reads were generated using Illumina NovaSeq 6000 platform.

### Alignment statistic and variant calling

Raw data files were quality checked in FastQC for each sample and sequence reads (FASTQ files) were later trimmed for adapters. Then aligned to the reference genome *Bubalus bubalis* (accession ID GCF_019923935.1 (www.ncbi.nlm.nih.gov)) using Burrows-Wheeler Aligner tool.^27^ Samtools were utilized to create BAM files^28^ thereby duplicate reads were removed using Samblaster.^29^ Per-base depth of coverage in the whole exome apprehended areas in each alignment file was assessed using depth of coverage utility of the Genome Analysis Toolkit (GATK).^30^

Base Quality Score Recalibration (BQSR) was carried out prior to variant calling using GATK’s BaseRecalibrator. GATK’s AnlyzeCovariates tool was used for recalibration around the InDels. Then, Haplotype Caller tool of GATK was used for calling the InDels under the usual recommendations for whole exome sequencing.^31^ CNNScoreVariants was used to score the filtered variants. We chose the InDels that met our quality control requirements, which included a call rate of more than 99% and a HWE with a P value more than 10^−4^.

### Linkage disequilibrium pruning of datasets and population structure analysis

By applying –indep-pairwise algorithm of plink (version 1.9),^32^ Linkage Disequilibrium (LD) pruning was implemented that filtered the LD areas having r^2^ values more than 0.1. It was performed using multiple sliding window widths to remove related markers to find the optimal parameters (the suggested admixture procedure sliding window size is 50 kb, and an extended 10,000 kb) for STRUCTURE and PCA analysis. For 10,000 kb window, r^2^ threshold of 0.1 to allow pruning of InDels was applied.

Admixture analysis was performed using STRUCTURE software (v2.3.4), that clusters using Bayesian approach^33^ in order to investigate admixture between the populations. After 20,000 burn-ins for 10 iterations, 30,000 MCMC runs were carried out, and the most frequent representation was chosen as the final instance of the population structure. PCA was utilized to investigate the genetic connection between studied breeds of buffaloes. PCA was performed using PLINK v1.9. For each population, the LD pruned data was used for PCA analysis. PCA plots were visualized by the ggplot2 package in R software.^34^

### Population genetic analysis

The average number of nucleotide changes per site on a population’s chromosomes is measured as nucleotide diversity. π = ∑_ij_x_i_x_j_π_ij_; where i,j €{1, …, n}, and n is the total number of sample sequences. Additionally, x_i_ and x_j_ stand for the i^th^ and j^th^ sequences respective frequencies, while π_ij_ stands for the amount of nucleotide changes per nucleotide site between the i^th^ and j^th^ sequences. π of each breed over the entire sequence was performed using VCFtools (in windows of 100 kb)^35^ and visualized using R software. To test the significance of the nucleotide diversity parameter for each of the population, one-way ANOVA (analysis of variance) was implemented. There is comparison between average number of pairwise nucleotide differences (θ_T_ = π) with the total number of segregating sites (θ_w_ = S/an), such that D = (θ_T_ - θ_w_)/√Var (θ_T_ - θ_w_), is known as Tajima’s D statistic.^36^ Tajima’s D analysis was performed using VCFtools, with the parameter “–Tajima’s D” (in windows of 100 kb).^37^

### Using exome-wide FST values, genetic differentiation analyses across populations

Finally, examination of population differentiation by estimating exome-wide F_ST_, using Weir and Cockerham method (1984), which is based on Wright’s F-statistics.^38^^,^^39^ F_ST_ is a descriptive statistic, extensively applied, and is executed to estimate genetic differentiation between populations under study.^2^^,^^3^^,^^40^ Previously, the weighted F_ST_ values were calculated for each variant using VCFtools with the parameter “–weir-fst-pop group1 –weir-fst-pop group2”.^41^ Here, group1 indicates the evaluation of F_ST_ for the particular population and group 2 comprises of remaining three breeds population. For each comparison, F_ST_ values were then averaged over sliding window of 100-kb along with step size of 25-kb. To detect accurately highly differentiating loci, F_ST_ values were Z-transformed as follows ZF_ST_ = ((F_ST_ - µF_ST_)/ΣF_ST_). Finally, the top 0.5% of windows with extraordinarily high F_ST_ values (top 0.5% of ZF_ST_ distributions) were candidate selection zones that probably subject to natural selection.

### Gene annotation, enrichment, and network analysis

Based on ZF_ST_ values, a gene was considered to have evidence of selection if it spanned an outlier genomic window. Genome annotation was done using NCBI’s Genome Data Viewer. Gene enrichment analysis was performed using PANTHER v.14.1.^42^ Examination of genes that were enriched under functional categories of GO-slim Biological Process term (BP) and panther pathways as being overrepresented by PANTHER using fisher’s exact test with false discovery rate correction at 5%. Gene network analysis was also used as a corresponding method to investigate how these reported genes were connected functionally by using STRING v11.^43^

## Results

### Sample collection, exome sequencing, and variant calling

Exome sequencing produced approximate 15 to 21 Gbp/sample of paired-end raw reads with a length of 159-bp from four buffalo breeds. Around 45.5–85.5 million clean paired-end reads were attained per sample per breed post quality control criteria (Table 1). Almost all the clean reads were mapped to the most recent buffalo reference genome (*Bubalus bubalis* (GCF_019923935.1)), which was around 98.50% of the total (Table 2).

**Table 1.** Summary of sequence data of all four buffalo breeds.

		Raw Reads			Trimmed Reads		
Breeds	Reads	Average GC (%)	Length (bp)	Total sequences (Millions)	Average GC (%)	Length (bp)	Total sequences (Millions)
Chhattisgarhi_1	R1	54	159	65.8	54	146	63.4
	R2	54	159	65.8	54	146	63.4
Chhattisgarhi_2	R1	54	159	86.7	54	147	83.5
	R2	54	159	86.7	54	147	83.5
Chhattisgarhi_3	R1	53	159	96.7	54	146	93.2
	R2	54	159	96.7	54	146	93.2
Chilika _1	R1	52	159	85	52	150	81.4
	R2	52	159	85	52	150	81.4
Chilika_2	R1	51	159	49.7	51	150	47.6
	R2	51	159	49.7	51	150	47.6
Chilika_3	R1	50	159	32.7	50	150	30.8
	R2	50	159	32.7	50	150	30.8
Chilika_4	R1	51	159	50.9	51	150	48.6
	R2	51	159	50.9	51	150	48.6
Gojri_1	R1	53	159	68.8	54	147	66.4
	R2	53	159	68.8	54	147	66.4
Gojri_2	R1	54	159	76.7	54	148	73.8
	R2	54	159	76.7	54	148	73.8
Gojri_3	R1	53	159	85.8	54	147	82.8
	R2	53	159	85.8	54	147	82.8
Murrah_1	R1	52	159	62.2	52	149	60
	R2	52	159	62.2	52	149	60
Murrah_2	R1	52	159	62	52	149	60
	R2	52	159	62	52	149	60
Murrah_3	R1	51	159	75.1	51	150	72.1
	R2	51	159	75.1	51	150	72.1
Murrah_4	R1	51	159	75.1	51	150	72.1
	R2	51	159	75.1	51	150	72.1

**Table 2.** Summary of sequencing and mapping results of all four buffalo breeds.

Sample	Total reads	Mapped reads
Chhattisgarhi_1	127014478	99.60%
Chhattisgarhi_2	167143508	99.00%
Chhattisgarhi_3	186567775	99.09%
Chilika_1	162948392	99.24%
Chilika_2	95355400	99.08%
Chilika_3	61663798	99.21%
Chilika_4	97368928	99.27%
Gojri_1	133068276	98.99%
Gojri_2	147901772	98.92%
Gojri_3	165897460	98.88%
Murrah_1	120117156	99.01%
Murrah_2	89003072	98.87%
Murrah_3	144494497	99.08%
Murrah_4	157245880	99.05%

**Figure 1.**
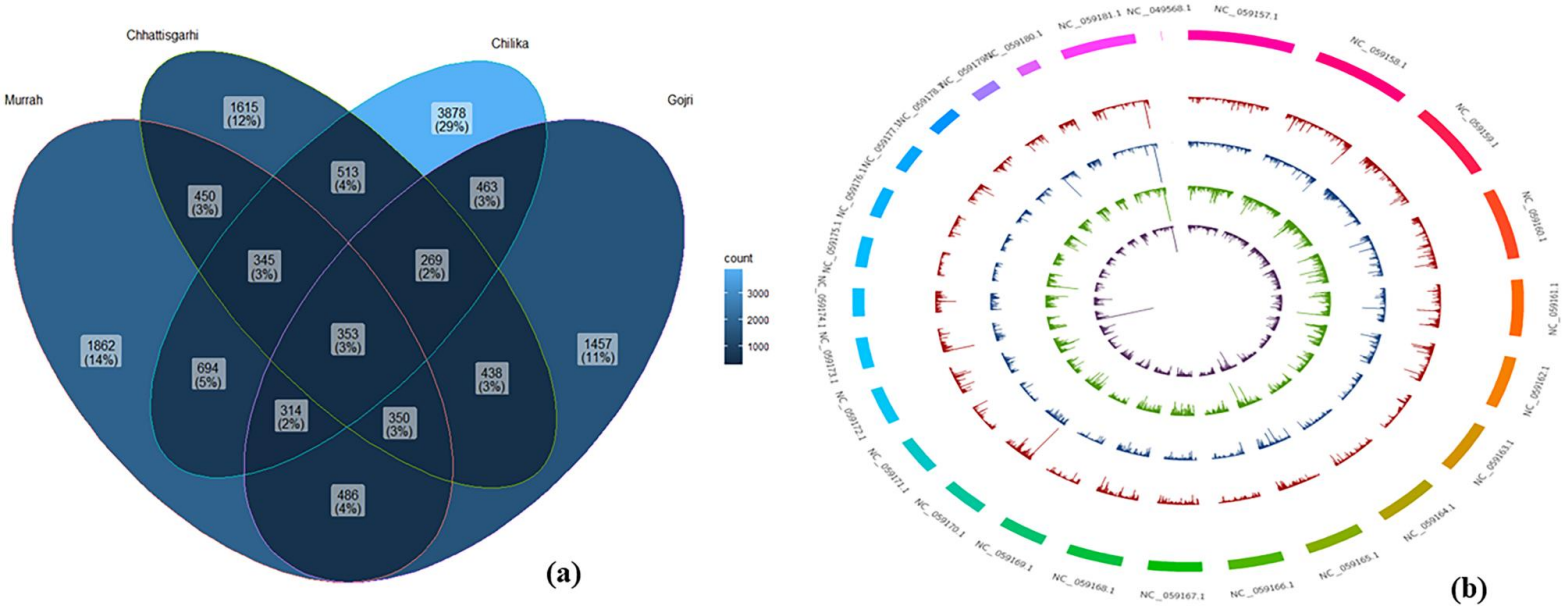
(a) Venn diagram depicting common and unique InDels in all four breeds of buffalo. (b) Exome-wide probability of InDels detection in four breeds of buffalo. The content of the buffalo reference genome sequence was represented by the outermost circle. Proceeding from outer to inner of the circle showed the exome-wide spreading of InDels in Chhattisgarhi, Gojri, Murrah, and Chilika, respectively.

Approximately, 63327 in Chhattisgarhi, 115765 in Chilika, 57371 in Gojri and 105306 in Murrah breed InDels were extracted. Based on the stringent thresholds, 4333, 6829, 4130, and 4854 InDels were retained for further processing in Chhattisgarhi, Chilika, Gojri, and Murrah breeds, respectively. The largest number (3878) of unique InDels was detected in the Chilika breed and Gojri breed displayed the lowest number (1457) (Figure 1a). The length of InDels ranged from −135 bp (deletion) to +130 bp (insertion) in Chhattisgarhi, −104 bp (deletion) to +137 bp (insertion) in Chilika, −116 bp (deletion) to +127 bp (insertion) in Gojri and −90 bp (deletion) to +104 bp (insertion) in Murrah breeds. However, maximum number of the identified InDels have the lengths less than 4 bp. Strikingly, high probability of detection of InDels was present on several chromosomes, such as on chromosomes 2, 14, and 17 in Chhattisgarhi, on chromosomes 4,5,6 and 23 in Gojri, on chromosomes 3, 4,5,9 and 19 in Murrah, and on chromosomes 6, 9, 17 and 18 in Chilika breed (Figure 1b).

### LD Pruning of datasets and population structure analysis

**Figure 2.**
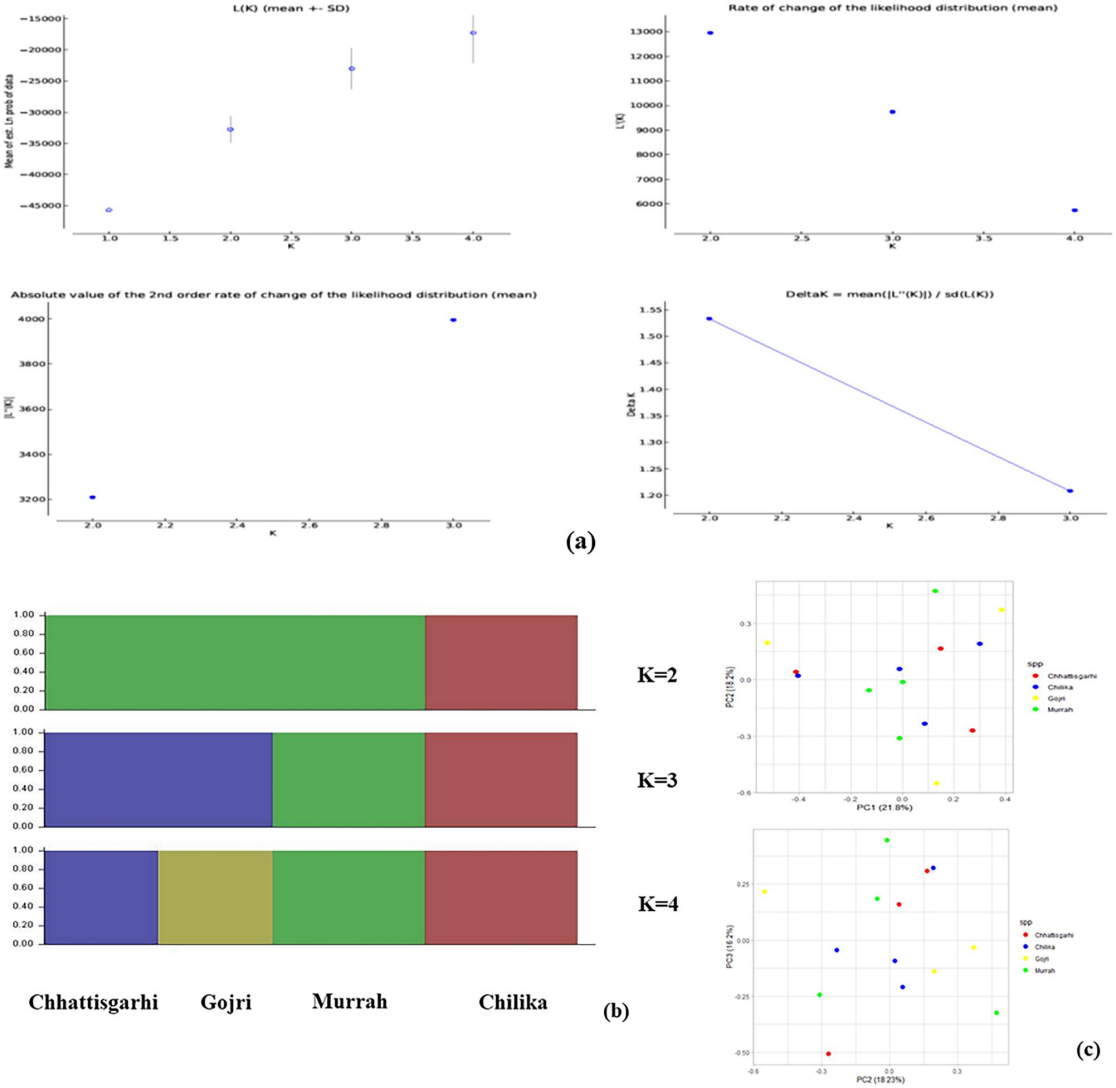
Population structure and relationships of Chhattisgarhi, Chilika, Gojri and Murrah buffaloes. (a) The optimal number of clusters present in the analyzed buffalo germplasms was estimated using the Evanno technique employing estimates of the number of sub-populations (K) using various statistics. (b) Buffalo breeds model-based clustering with K = 2 to 4 using the STRUCTURE programme. (c) Using principal component analysis (PCA), the comparison of PC1 and PC2 and PC3 is shown.

Around 442, 769, 420, and 542 InDels were retained in Chhattisgarhi, Chilika, Gojri and Murrah breeds post LD pruning. A maximum of three populations were proposed by the number of potential sub-populations assessed using the Evanno K technique, where modal value of delta K at true K was missing (Figure 2a). Genetic structure of all the four breeds was inferred by STRUCTURE analysis (Figure 2b). At K = 2, Chilika breed clustered separately, while rest of three populations were grouped into single population. At K = 3, Murrah clustered separately from the other two breeds that were grouped as single population. At K = 4, all four populations were genetically distinct and clustered into four different breeds without any admixture. PCA result illustrates a more scattered pattern of all four breeds. The first eigenvector (PC1) explained 21.8% and subsequently PC2 and PC3 explained 18.2% and 16.2% of the total variance, respectively (Figure 2c).

### Population genetic analysis

**Figure 3.**
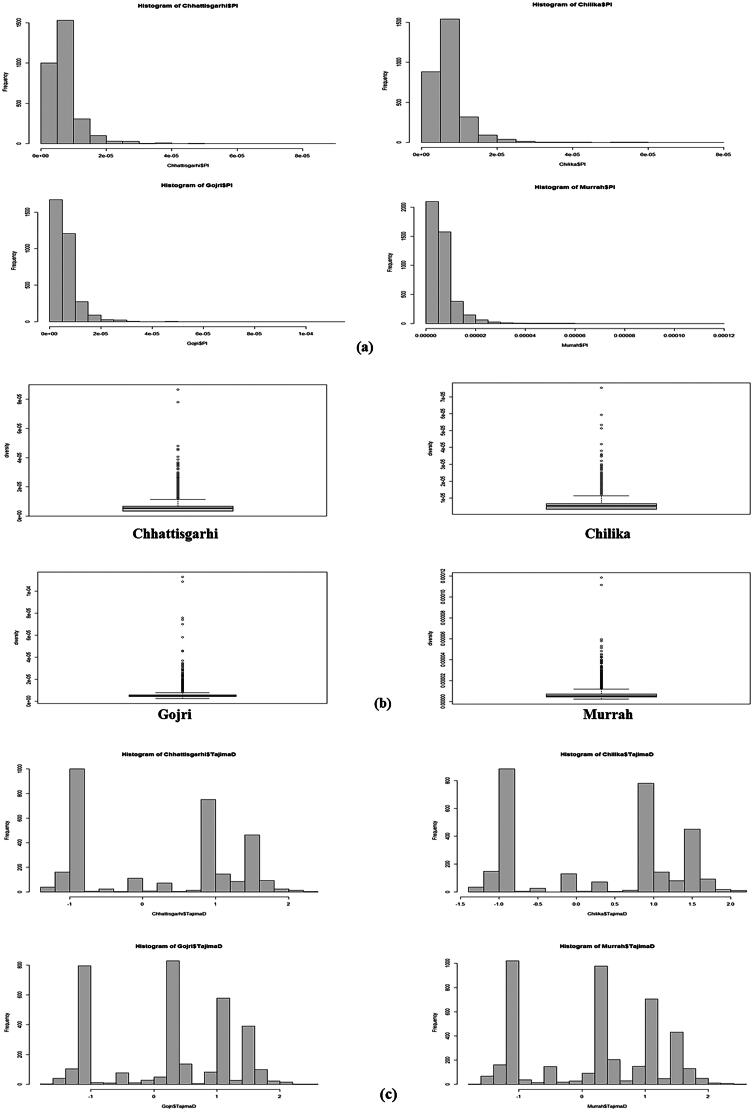
Summary statistics for population genetics. (a) Histogram showing frequency of different nucleotide diversity values throughout chromosome. Summary statistics for population genetics. (b) Box plots displaying distribution of nucleotide diversity, measured exome-wide across 100-kb windows in all four breeds. (c) Histogram showing frequency of different Tajima’s D values throughout exome.

Average nucleotide diversity of Chilika, Chhattisgarhi, and Murrah was significantly higher in comparison to Gojri population (*p* < 0.01) (Figure 3a and b). However, the average nucleotide diversity of Chilika, Chhattisgarhi and Murrah was significantly similar (*p* < 0.01) (Table 3). Chromosome-wise analysis depict maximum nucleotide diversity on chromosome number 18 and 23 in Chhattisgarhi, on chromosome number 18 in Chilika, on chromosome number 12 and 18 in the Gojri breed, and chromosome number 21 in Murrah. Mean values of TD were around zero for all four breeds (Figure 3c) suggesting populations were under slight balancing selection. Chilika buffalo showed more positive value (TD = 0.267374), followed by Gojri (TD = 0.25392), subsequently by Chhattisgarhi (TD = 0.22114) and least showed by Murrah (TD = 0.21571).

**Table 3.** The summary of nucleotide diversity in all four buffalo breed populations.

Breeds	Nucleotide diversity
	Mean ± SE
Chhattisgarhi	0.000006692 ± 0.000000094^b^
Chilika	0.000006694 ± 0.00000009^b^
Gojri	0.000006152 ± 0.000000095^a^
Murrah	0.000006557 ± 0.000000087^b^

Note: Different capital letters in the same column indicate significant differences between populations (*p* < 0.01), while the same letters indicate no significant differences.

### Genetic differentiation analysis across populations using global F_ST_ values

**Figure 4.**
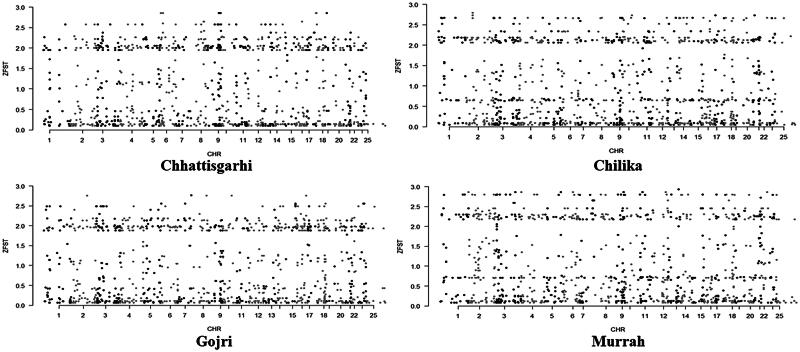
Whole exome distribution of ZF_ST_ values estimated in 100-kb sliding windows with 25-kb increments in all four breeds of buffalo.

To minimize false positives, only the top 0.5% of windows with high ZF_ST_ values were identified as distinguishing areas in the empirical distributions for each breed. Therefore, 27, 98, 38, and 35 outlier windows (ZF_ST_ ≥ 2.64, 2.67, 2.55, and 2.86; corresponding F_ST_ ≥ 0.343, 0.334, 0.343, and 0.342) were detected in the Chhattisgarhi, Chilika, Gojri and Murrah buffalo, respectively (Figure 4). Comparing their outlier windows, it was depicted that most of these differentiating loci were breed-unique, portraying different phenotypic evolutions under adaptations to the local environments. Summary of identified genes for various performance traits under the studied breeds is depicted in Table 4.

**Table 4.** Summary of genes identified for performance traits in all four studied breeds.

Breeds	Performance traits					
	Milk yield	Milk composition	Fertility	Adaptation		
				Salinity	High-altitude	Immunity
**Chhattisgarhi**	*CAMSAP3*	*CLCN1, CASP2*				
**Chilika**		*NUDT3*		*RPS28,* HMGA1, *GRM4, KCNA3*		*LCN2*
**Gojri**			*DBI*		*APBB2, TH, CTU1, ALKBH8, INS,* IGF2, *CALD1, HABP2*	
**Murrah**		*LPAR6, FADS1*	*PTGER3, PARVG*			*ITGB1BP1*

### Gene annotation, enrichment, and network analysis

**Figure 5.**
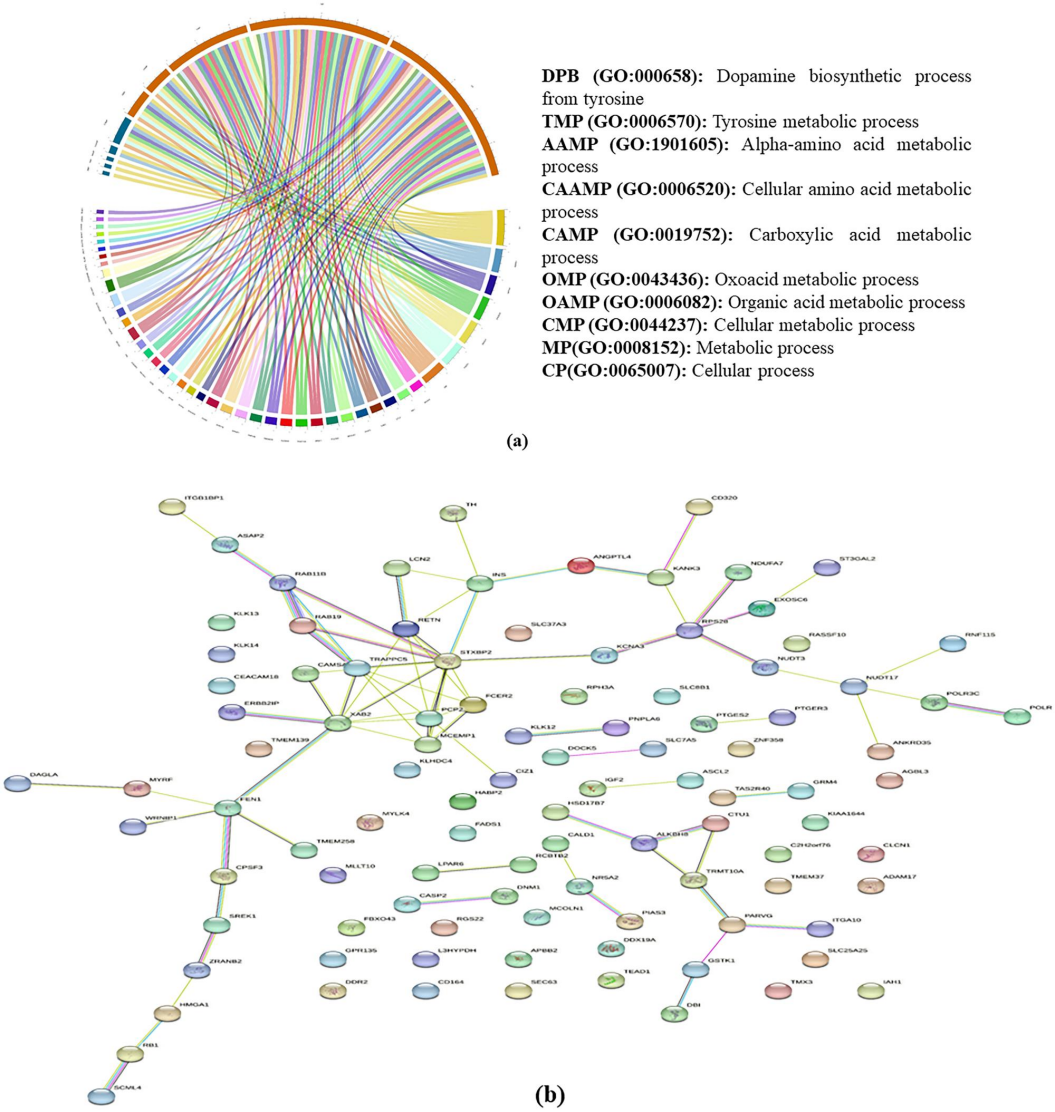
(a) Summary of the top 10 GO-slim biological processes into which the candidate genes were classified. (b) Network analysis of genes identified in present study in all four breeds.

After the genome annotation, a total of 38 (Chhattisgarhi), 37 (Chilika), 23 (Gojri), and 29 (Murrah) genes were found in the putative outlier windows of each breed. However, under GO-slim biological processes and panther pathways, none of the genes were significantly enriched, after correcting for fisher’s exact test. These genes were classified into many biological processes, top 10 biological processes like the dopamine biosynthetic process (GO:000658) metabolic process (GO:0008152), cellular process (GO:0065007), etc., is presented in Figure 5a. Using STRING database, from annotated 127 genes, 41 were found to be clustered in the main network (Figure 5b). The 41 genes that make up the primary gene network were identified by the centrality analysis as having the most connections with various other candidate genes. Table 5 depicts top 10 genes showing maximum number of interactions. Based on the results of population structure and differentiation, biological functions of the genes, and data from research that have been published, many genes were potentially accountable for the performance attributes in these unique buffalo breeds and are presented in greater detail below.

**Table 5.** Top 10 genes based on the number of interactions identified in the STRING database.

Gene symbol	Number of interactions
*STXBP2*	10
*XAB2*	9
*TRAPPC5*	8
*PCP2*	7
*FCER2*	6
*RETN*	6
*INS*	5
*RPS28*	5
*FEN1*	5
*MCEMP1*	4

## Discussion

Buffaloes have spread widely around the world as a result of human migration and the growth of agricultural commerce, especially in Asia and developing nations like India. They can endure tough environments and circumstances while assisting low- and middle-income farmers in maintaining their way of life.^44^ Present study was laid with objective to explore large number of variants and genes that are under long-term natural selection for performance traits that will provide valuable genetic resource for improvement in these breeds in future. This is the foremost whole-exome based comparative study including diverse individuals from all four breeds. For population genetic analysis, with decreasing sequencing cost, ddRAD is now almost obsolete as it results in much missing data depicting biased results.^45^ Pre-selected SNPs in the array for the BEADCHIP dataset may not fundamentally be a uniform representation of the populations under study. Also, an enlarged percentage of ancestry informative markers in the data will estimate biased results for statistics related to population genetic studies.^25^ With whole genome data, the identification of lot many uninformative variants (neutral or weak effect) along with the associated cost and complexity of data analysis makes its accessibility limited.^46^ Foremost, whole exome data is not affected by any of the biases discussed above and is most preferred for population based genetical studies.^24^^,^^25^^,^^47^

Wider prospective of genome understanding of how the natural selection has shaped the pattern of genetic variation that not only explores insights into mechanism of evolutionary changes but also sightsee population structure associated with complex traits that further elucidate genetic evaluation, environmental adaptation, and protection of the genetic resources.^48^^,^^49^^,^^50–53^ So far limited studies in Chhattisgarhi, Chilika, Gojri, and a few in Murrah have been carried out for performance attributes.^6^^,^^9^^,^^10^^,^^54^^,^^55^ The most significant qualities of buffalo are their performance aspects, which are also among their most significant economic traits. Quantitative qualities such as performance are regulated by polygenes.^56^ In a DNA sequence, insertions and deletions (indels) are additions or deletions of one or more nucleotides. InDels cause similar levels of variation as SNPs and have widely variable effects on gene expression and thus can modify the function of genes.^57^ InDels are thought to encompass more polymorphic base pairs than SNP.^58^^,^^59^ InDels are being widely used in population genetics studies.^60–62^

In present study, 3 to 4 samples per breed were taken ensuring sample collection was from different parts of their entire breeding tract. Reportedly, very small sample size of n = 2 is optimum to get precise estimate of F_ST_ in population genomics of non-model organism using NGS markers.^63^ Small sample sizes (n = 3 to 4) of random individuals for particular breed are required to yield reliable estimates of differentiation with high precision when using high-throughput markers.^64^^,^^65^ Limited sample size in present study did not only reduce the cost of our research project but also enabled us to analyze greater variety of populations at the same price. Further, it provided a significant benefit in population genetic research on breeds like the Chilika whose sample collection is tedious.

The foundation of certain population genetic methods, like admixture analysis and PCA, is the premise of linkage equilibrium. Due to the fact that closely connected markers cause some haplotypes to occur more frequently than predicted due to LD and because huge blocks of markers in complete LD can significantly affect the eigenvector/eigenvalue structure, it is crucial to remove linked markers from datasets.^66^ This is crucial for the exome dataset in particular since genes may be crammed into tiny, transcriptionally active euchromatin areas of the chromosome. Modal value of delta K was vague at true K. This might be due to less sample size used in our study. Later, STRUCTURE analysis clearly distinguished Chilika from other population at K = 2, indicating Chilika buffalo have unique evolutionary features. Chilika is primarily riverine but few hybrids (riverine x swamp) were also reported in the region.^67^ This might be the probable reason that Chilika got separated from the other three riverine breeds. The rest of the 3 populations might have a closer ancestral relationship so were assigned to one population. At K = 3, Murrah separates itself, and there was no genetic differentiation between Chhattisgarhi and Gojri breeds. At K = 4, STRUCTURE assigned all four populations into four different breeds without any admixture. This indicated that studied populations were quite differentiated. Probable reason for this might be due to their different geographical locations, living environment of breeds, and long-term natural mating state, with minimal artificial selection apart from Murrah breed. Our result of clear genetic differentiation between Gojri and Murrah breed was consistent with previous studies where Gojri breed was having pure blood of 99.4% of Gojri with 0.4% of admixture from Murrah breed, indicating Gojri is a rare breed with high genetic variability.^55^ To further elucidate the genetic relationship between studied populations, PCA was explored. The total variance explained by all three eigenvectors was around 56.2%, which substantially contributes to variation. Because each breed sample was taken from different regions of their breeding tract, it is possible that the PCA analysis of these breeds showed a more scattered pattern indicating the more genetic distance between individuals of the same breed.

In present study, Gojri showed a reduced level of nucleotide diversity compared to other three breeds. This might be probably due to reason that individuals of Gojri are more genetically related and thus within population genetic diversity is less. Rest three breeds had almost similar level of nucleotide diversity that might be likely influenced by natural and environmental selection in each region, thus resulting in different genomic characteristics. TD statistics result depicts the presence of slight balancing selection in all breeds population. The slight balancing status in the studied population might be probably elucidated by the nonoccurrence of any breeding related program in Chilika, Chhattisgarhi, and Gojri during the past years. Similar results were shown in Mangalarga Marchador horses that were under strong balancing selection.^67^ After the above analyses, unequivocal evidence of pronounced genetic variation exists in the breeds under study. Thus, further F_ST_ statistics was explored for population differentiation associated with performance traits. Despite the possibility of false positive results from genome scans based on population differentiation, such as F_ST_, this technique is most effective for identifying differentiating loci in populations with unknown phenotypes.^68–71^ Several breed-specific unique and highly differentiating loci were identified in the present study.

### Candidate genes associated with milk yield

Genomic region encompassing *CAMSAP3* (Calmodulin regulated spectrin associated protein family member 3) (ZF_ST_=2.85) was found to be involved in a variety of GO-slim BP including cellular component organization (GO:0022613), complex of protein subunit organization (GO:0043933), ribosomal large subunit biogenesis, etc., in Chhattisgarhi breed. Previous studies suggested that *CAMSAP3* was found to be related with milk yield in Egyptian buffaloes.^72^ *CAMSAP3* played central role impacting the lactation process in *Bos taurus, Ovis aries, and Bubalus bubalis*.^73^ Average milk yield was reported to be 4 kg per day with 7 to 12 months of lactation length in Chhattisgarhi.^11^ *CAMSAP3* could be one probable reason for minorly affecting milk yield in this breed.

### Milk protein associated candidate gene

Whey protein and casein make up the majority of the milk proteins, and their synthesis is controlled by a number of hormones and biological processes. Several genes were identified as differentiating genomic regions under natural selection. Among these, *CLCN1* (Chloride channel protein) (ZF_ST_=2.64) encodes ion channel, *CASP2* (Caspase 2) (ZF_ST_=2.64) encodes protease in Chhattisgarhi, *NUDT3* (Diphosphoinositol polyphosphate phosphohydrolase 1) (ZF_ST_=2.73) encodes phosphatase in Chilika and *LPAR6 (*Lysophosphatidic acid receptor 6) (ZF_ST_=2.93) encodes G protein-coupled receptor in Murrah. These genes were enriched under GO-slim BP like cellular process (GO:0009987). *CASP2* and *CLCN1* both were informed to be related with protein yield in milk of Holstein bovine of North American.^74^ These genes might be related with increase in nutritional quality of milk in Chhattisgarhi.

Previous study reported that *NUDT3* responsive mRNAs were discovered to have a role in cell motility, and the stability of integrin 6 and lipocalin-2 mRNAs was directly correlated with NUDT3 decapping.^75^ *NUDT3* was previously associated with 305-day yield of milk by impacting protein of milk production through its involvement in mRNA decay in Holstein cows.^76^ There is a huge demand for milk of Chilika due to its nutritional qualities, and most probable reason might be they solely feed on natural vegetation that are highly nutritionally enriched. This gene could also be one of the reasons for promoting nutritional value of Chilika milk. *LPAR6* gene was identified to be the most promising gene that influences milk protein content in Chinese Holstein.^77^ Therefore, *LPAR6* might be most likely responsible for milk protein percentage in Murrah buffaloes.

### Candidate genes associated with milk fat

Milk contains 99% milk triglycerides, a superior natural fat that is crucial to milk’s composition.^78^ Genomic region differentiated in Murrah breed encompassed *FADS1* (fatty acid desaturase 1) (ZF_ST_=2.86). FADS1 adds double bond at 5^th^ and 6^th^ position of long-chain polyunsaturated fatty acids in Canadian Holstein cattle’s milk.^79^ This gene in accordance with previous studies, might be related with milk fat yield in Murrah.

### Reproduction trait associated candidate genes

Several genes were recognized in highly differentiating exonic regions under selection. These were enriched to various GO-slim BP like cell morphogenesis involved in differentiation, developmental process, cellular process, cell-substrate adhesion, actin cytoskeleton reorganization, etc. Among these, *DBI* (Acyl-CoA-binding domain-containing protein 5) (ZF_ST_=2.76) encodes transfer/carrier protein in Gojri and *PTGER3* (Prostaglandin E2 receptor EP3 subtype) (ZF_ST_=2.86) encodes G-protein coupled receptor and *PARVG* (Parvin gamma) (ZF_ST_=2.86) encodes actin and actin related protein in Murrah breed. Previous studies reported DBI gene was involved in the regulation of mitochondrial steroidogenesis and was predominantly found immunodetected in cells with secretory activity, such as hepatocytes, steroid-producing cells in the adrenal cortex, and testis, etc in mammals.^80^ *PTGER3* gene was related to fertility traits in bovines^81^ and *PARVG* gene was associated to a number of viable embryos in dairy cattle.^82^ These genes might be related with reproductive processes in Gojri and Murrah breeds.

### Candidate genes associated with saline adaptation in Chilika buffalo

Genomic regions encompassing few genes that were enriched to GO-slim BP like regulation of biological process, chemical synaptic transmission modulation, cellular processes, ion transmembrane transport, potassium ion transmembrane transport, etc. *HMGA*1 (High mobility group AT-hook 1) (ZF_ST_=2.73) encodes endodeoxyribonuclease, *GRM4* (Glutamate metabotropic receptor 4) (ZF_ST_=2.67) encodes G-protein coupled receptor, and *KCNA3* (Potassium voltage-gated channel subfamily A member 3) (ZF_ST_=2.67) encodes voltage-gated ion channel in Chilika breed. *HMGA*1 reported to affect human height and making it feasible for certain anthropometric features like adult height.^83^^,^^84^ Chilika has the unique ability of a behavioral pattern of walking into the marshy area without any difficulty.^54^ *HMGA1* might be responsible for anthropometric features adapted to walking into marshy lands. *GRM4* was earlier said for regulating mineral content in the muscle of Nellore cattle.^85^ Normal effects of muscle mineral composition on nerve impulse transmission, osmotic pressure, bone and skeletal tissue function, and the preservation of acid-base equilibrium. *GRM4* may be responsible for such homeostasis under saline conditions. *KCNA3* was previously reported to be associated with adaptation to alkaline waters in Amur Ide.^86^ This gene might be one of the reasons for homeostasis and osmoregulation in the Chilika breed inhabiting saline conditions.

### Candidate genes associated with a longer shelf life of Indian Chilika curd

Highly differentiating genomic region encompassing *RPS28* (40S ribosomal protein S28) (ZF_ST_=2.73) that encodes for ribosomal protein was identified in Chilika breed. This gene was enriched for GO-slim BP like cellular aromatic compound metabolism, RNA metabolism, and nucleobase-containing chemical metabolism, etc. Earlier studies suggested that *RPS28* gene expression pattern recommends the possible higher antioxidant activity of milk in Kashmiri cattle.^87^ Dairy products like curd and yogurt derived from the milk of the Chilika breed have a longer shelf life. primarily, Chilika curd possesses beneficial microflora like Lactobacillus, Lactococcus, Streptococcus, Leuconostoc, and yeast which shows anti-fungal activity by the secreting number of anti-fungal compounds such as 3- hydroxyl fatty acid, caproic acid, etc. Subsequently, Chilika curd is enriched with thermo-tolerant lactobacilli inoculum, Dendrocin- an antifungal protein isolated from a fresh bamboo shoot.^14^^,^^88^ This gene might also be one of the reasons for imparting longer shelf life of the curd prepared from milk of Chilika buffalo.

### Candidate genes associated with high altitude adaptation

Few differentiating genomic regions that were under selection were enriched for GO-Slim BP like cellular process, tyrosine metabolism, catecholamine metabolism, cellular biogenic amine metabolism, organic substance metabolism, etc. *APBB2* (Amyloid beta precursor protein binding family B member 2) (ZF_ST_=2.55), *TH* (Tyrosine 3-hydroxylase) (ZF_ST_=2.55) encodes oxidoreductase and *CTU1* (Cytoplasmic tRNA 2-thiolation protein 1) genes in Gojri buffalo were retrieved from this part of exome. Few genes were previously reported to be associated with altitude adaptation namely *ALKBH8* (Alkylated DNA repair protein alkB homolog 8) (ZF_ST_=2.76), *INS* (insulin) (ZF_ST_=2.55) encode growth factor, *IGF2* (insulin-like growth factor 2*)* (ZF_ST_=2.55)*, CALD1* (Caldesmon 1) (ZF_ST_=2.76)*, HABP2* (Hyaluronan Binding Protein 2) (ZF_ST_=2.55) were also identified in our study located to this region of genome in Gojri. *ALKBH8* controls the expression of selenocysteine-protein to prevent damage from reactive oxygen species in mice (ROS).^89^ Low oxygen pressure and an increase in the production of reactive oxygen are caused by high altitude exposure, and these changes are frequently accompanied by an increase in oxidative damage to lipids, proteins, and DNA. Gojri are semi-migratory and move to top hills to avoid peak summers of plain areas. Change in altitude from plain to hills might leads to low oxygen pressure which ultimately generates ROS.

*ALKBH8* gene might be probably responsible for preventing any damage caused by free radicals. *INS* gene regulates insulin biosynthesis in pancreatic beta cells in humans.^90^ *IGF2* was reported to encode an essential growth factor and plays an important role in muscle growth in different developmental stages in Chinese Qincuan beef cattle breed.^91^ *CALD1* expressed in smooth muscle, it inhibits vascular smooth muscle tone in people, regulates smooth muscle contraction.^92^ *HABP2* was related to cell growth and proliferation in Brazilian Nellore beef cattle.^93^ *TH* was reported to be activated in the adrenal medulla during the time of prolonged stress in bovine adrenal medullary cultured cells.^94^ *CTU1* was said to have an impact on both production and non-production qualities, making it a crucial marker for a dairy cattle selection.^95^ *APBB2* involved in amyloid precursor protein processing and involvement in cell cycle regulation in different cells lines.^96^ Dry and non-pregnant Gojri, migrate from the plains/foothills of Punjab and Himachal Pradesh to the top hills of Kangra and Chamba divisions of Himachal Pradesh. Typically, it takes them 10 to 15 days to finish their voyage. Highland has a frigid climate, which is feasible for buffaloes to manifest symptoms of heat and have a high conception rate. Most buffaloes used to be pregnant when they traveled back. The migratory path is extremely undulating and challenging to traverse. Its most difficult to avoid toppling down and slipping when pregnant and moving downhill. These buffaloes have the peculiar feature of “sure footing”. This makes them walk and graze hilltops without slipping. Identified genes might be responsible for the development and strengthening of muscles of limbs and other body parts enabling laid-back mobility even during pregnancy.

### Candidate genes related to immunity

Two genes were identified to be under natural selection, *LCN2* (Neutrophil gelatinase-associated lipocalin) encoding transfer/carrier protein in Chilika and *ITGB1BP1* (Integrin beta-1-binding protein 1) (ZF_ST_=2.86) in Murrah. These were enriched for the pathways like the control of cellular processes, the organization or biogenesis of cellular components, etc. *LCN2* reported to act as a natural bacteriostatic agent against bacterial infection and inflammatory conditions in mammals.^97^^,^^98^ Chilika has a very low mortality rate without any medications. This gene might be one of the most probable causes of low mortality in this breed. *ITGB1BP1* reportedly associated with disease immune mechanisms and adaptation to the tropical environment in Nigerian cattle.^99^ This gene might be related to the adaptation of Murrah buffaloes to tropical climatic conditions and resistance to various diseases occurring in such climates.

Further, gene to gene interaction indicated that *NUDT3* interacted (confidence score = 0.613) with *RPS28* (increase in the shelf life of curd candidate) gene. *INS* interacted with a confidence score of 0.416 with the *TH* gene and with a confidence score of 0.424 with the *LCN2* candidate gene. *KCNA3* interacted (confidence score = 0.559) with the *RPS28* candidate gene. *KCNA3* is a voltage-gated ion channel gene. *ALKBH8* interacted with a confidence score of 0.953 with the *CTU1* candidate gene. *ALKBH8* gene prevents free radical-based damage to the cells. This suggests that most of identified genes might have interrelated functions. Few genes were interacted with one or two or with none of the genes suggesting these genes might have independent functions or might be interacting with the other genes that are not included in this interaction.

## Conclusion

Exome sequencing, which is preferred for population genetic analysis has provided evidence of overall patterns of genetic structure and differentiation, dissecting apart the genetic variants and genomic regions showing signals of natural selection specific to each of the studied breeds. Exome sequencing and analysis unmasked several genes that may have role in the regulation of performance and adaptation traits in the studied rare (Gojri, Chilika and Chhattisgarhi) buffalo breeds. These genes can be further validated with candidate gene approach to establish their roles in regulating the traits. The results of this study will enable breeders and geneticists in planning breeding and conservation programs in Chhattisgarhi, Chilika, and Gojri that later will pave the way for establishing the set of genomic markers which can be utilized in execution of genomic selection and breeding in the studied breeds.
